# Weight cycling based on altered immune microenvironment as a result of metaflammation

**DOI:** 10.1186/s12986-023-00731-6

**Published:** 2023-02-22

**Authors:** Wanyang Li, Wei Chen

**Affiliations:** Department of Clinical Nutrition, Chinese Academy of Medical Sciences - Peking Union Medical College, Peking Union Medical College Hospital, No. 1 Shuaifuyuan, Dongcheng District, Beijing, 100730 China

**Keywords:** Weight cycling, Metaflammation, Immune cell, Adipokines, Oxidative stress

## Abstract

As a result of the obesity epidemic, more people are concerned about losing weight; however, weight regain is common, leading to repeated weight loss and weight cycling. The health benefits of early weight loss are nullified by weight regain after weight cycling, which has much more severe metabolic consequences. Weight cycling alters body composition, resulting in faster fat recovery and slower muscle reconstruction. This evident fat accumulation, muscle loss, and ectopic fat deposition destroy the intestinal barrier, increase the permeability of the small intestinal epithelium, and cause the lipotoxicity of lipid metabolites and toxins to leak into extraintestinal tissues and circulation. It causes oxidative stress and hypoxia in local tissues and immune cell infiltration in various tissues, all contributing to the adaptation to this metabolic change. Immune cells transmit inflammatory responses in adipose and skeletal muscle tissue by secreting cytokines and adipokines, which mediate immune cell pathways and cause metaflammation and inefficient metabolic degradation. In this review, we focus on the regulatory function of the immunological microenvironment in the final metabolic outcome, with a particular emphasis on the cellular and molecular processes of local and systemic metaflammation induced by weight cycling-induced changes in body composition. Metaflammation in adipose and muscle tissues that is difficult to relieve may cause weight cycling. As this chronic low-grade inflammation spreads throughout the body, metabolic complications associated with weight cycling are triggered. Inhibiting the onset and progression of metabolic inflammation and enhancing the immune microenvironment of adipose and muscle tissues may be the first step in addressing weight cycling.

## Introduction

Worldwide, the prevalence of overweight and obesity is unstoppable [1]. In 2016, more than 1.9 billion adults over 18 were overweight, and more than 650 million were obese [2]. In 2020, 39 million children under the age of five will be overweight or obese, and in 2022, the WHO estimates that approximately 60% of European adults will be overweight or obese [3]. It is currently one of the leading causes of health concerns among citizens in most nations [4]. Obesity is associated with cardiovascular disease, diabetes, chronic obstructive pulmonary disease, arthritis, and cancer [5–7]. Due to their high mortality rate, morbidity, and economic loss, obesity, and overweight have received considerable attention [8]. More than 40% of adults in the general population attempt to lose weight over five years, and 23% of adults report attempting to maintain their weight annually [8]. However, less than 20% of adults who attempt to lose weight can maintain a 10% weight loss for a year [9]. Consequently, obese individuals experience frequent weight fluctuations. According to a survey, 20% to 30% of adults engage in weight cycling (WC), which consists of cycles of weight loss (≥ 5 kg) and regain [10]. Although the effect of WC on future metabolic health is still debatable, there is abundant evidence that it poses certain health risks. It increases the risk of type 2 diabetes and strongly predicts cardiovascular disease characteristics [11–14]. In addition, it may increase the risk of certain tumors (e.g., renal cell carcinoma, endometrial carcinoma, and non-Hodgkin 's lymphoma) [15]. WC may increase future weight gain, alter body composition, and be associated with decreased muscle mass and strength [16, 17]. The decrease in lean body mass and increase in body fat mass reduces the basal metabolic level, which is related to immunological adaptation-induced metaflammation [18, 19]. The destruction of metabolic homeostasis due to excess energy may cause metaflammation, an adaptive response to relieve the anabolic pressure caused by obesity, and chronic low-grade inflammatory responses [20]. This inflammatory response may be permanent [21]. Consequently, this study aims to investigate the factors, sources, and molecular mechanisms of WC at three levels (systemic, tissue, and cellular) in terms of metaflammation during WC (Table 1).

**Table 1:** Systemic, tissue and cellular molecular correlates of WC

		Metabolic inflammatory factors associated with WC	Reference
System	Endocrine system	Adaptive response to fat recovery and more difficult weight loss; increase in postprandial blood glucose; increased risk of diabetes	[22–25]
	Circulatory system	An increase in blood pressure; deterioration of blood lipid levels in women; increased risk of fatty liver	[26–30]
	Intestinal barrier	Disruption of the intestinal barrier, including inhibition of mucus production, weakening of tight junctions, changes in the structure of the intestinal villi and intestinal inflammation; enlargement of the intestinal absorption area; increased secretion of pro-inflammatory cytokines in the gastrointestinal tract and down-regulation of intestinal immune defences; increased distribution of bacteria in the intestinal flora and leakage of pathogenic components and metabolites through the intestinal lumen; leakage of lipopolysaccharides and other toxins into the blood, causing metabolic endotoxaemia	[31–36, 39–44]
Tissue	Adipose tissue	More pronounced proliferation of preadipocytes; increased volume and number of adipocytes and accumulation of fat; increased adipose tissue cell pressure	[54, 59–63, 65–67]
	Skeletal muscle	Ectopic aggregation of lipids in skeletal muscle and formation of perimuscular adipose tissue; loss of muscle mass; decreased uptake of glucose transporter protein 4 (GLUT4); inhibition of mitochondrial function and increased formation of reactive oxygen species	[70–80]
Cellular and Molecular	Immune cells	Immune cell accumulation: B cells are the first to accumulate in adipose tissue and have a significant inflammatory-promoting effect on adipose tissue expansion, releasing IL-6 and interferon; subsequently the number of CD4 + and CD8 + T cells increases T cells; the number of macrophages gradually increases, IL-6 and TNF-α secretion increases, and the increase in circulating glucose and fatty acid substrates, lipotoxicity and tissue hypoxia, causing M1 macrophage polarization that persists	[85–89, 93, 97]
	Molecular Mechanisms	1. Immune cell activation: blockade of RIPK1 increases iNKT cell activity; a mixture of palmitic acid or long-chain fatty acids increases the expression of pro-inflammatory molecules in macrophages and induces M1 polarization through the involvement of TLR2 and TLR4 and activation of the JNK pathway; induces activation of the NLRP3 inflammasome, promotes maturation and secretion of IL-1β and IL-18, triggers oxidative stress and inflammatory response; Lmot4-resistin signaling presents adipose tissue and liver crosstalk	[53, 98-102, 104, 106, 111, 112, 115-117, 119, 121, 124, 125]
		2. Oxidative stress: adverse effects on 5-OHmU levels and significant oxidative damage; stimulation of the HIF-1 pathway triggers lactate accumulation and local inflammation; increased ROS levels and activation of metabolic inflammation	
		3. Adipokines: affects adipokine levels and affects macrophage polarization; decreased expression of lipocalin and resistin; dysregulated expression of CTRP3 causes increased inflammation and induces metabolic dysfunction; leptin promotes the release of pro-inflammatory cytokines; decreased serum leptin levels; increased levels of resistin and upregulated expression of stat3-activated IL-1β, IL-6 and IL-8; Lower levels of adiponectin stimulate macrophage inflammation	

## Systemic effects on WC: metaflammation of the endocrine system, circulatory system, and intestinal barrier

In the event of weight regain(WR) in the WC, i.e., a return to overweight or obesity, the health risks to systemic systems would be significantly increased. However, in the opposite direction, the metaflammation brought on by these systemic diseases is linked to weight gain. Therefore, this may be a two-way effect of systemic metaflammation influencing the weight and the cycle, causing systemic metabolic disease.

### Endocrine system and WC

Uncertainty surrounds the relationship between WC and metabolic diseases, which may affect systemic inflammation [22]. After an energy gap occurs during weight loss, there is a continuous increase in hunger and a decrease in energy consumption. Dietary changes significantly reduced the basic energy consumption of WC mice compared to long-term obese mice, which led to a rapid recovery of body weight and increased food intake after the calorie restriction period. It was also discovered that after multiple WCs, the weight loss was less than that of long-term obese mice [23]. Due to these adaptive responses to body fat recovery, there is an increase in glucose, triglycerides, and fatty acids in the postprandial circulation [24]. After weight restoration, animals with WC had higher fasting glucose levels and impaired glucose tolerance. Our review of animal studies suggests that the health benefits of diet-induced weight loss are not sustained after the weight is regained and that WC leads to adverse metabolic outcomes [25]. In population studies, WC has also been a strong independent predictor of newly diagnosed diabetes. Future research is required to examine the causal relationship between WC and diabetes risk [11].

### Circulatory systems and WC

According to studies, the number of weight cycles is not only positively correlated with systolic blood pressure and fasting blood glucose levels. However, it may also increase the risk of cardiovascular disease [26]. Additionally, WC reinforces cardiovascular risk factors (such as blood pressure, heart rate, sympathetic activity, blood glucose, fat, and insulin) that may repeatedly exceed normal values during weight recovery, thereby increasing cardiovascular stress [27]. In addition to fat gain and muscle loss, weight recovery affects the lipid levels in peripheral blood. Blood lipid levels were lower in women with WC than in those who maintained weight [24]. The incidence of hepatic steatosis was significantly higher in WC mice than in obese mice [28]. The pathological findings revealed that lipid droplets had entered the liver cells, and the levels of aspartate aminotransferase (AST) and alanine aminotransferase (ALT) increased, leading to liver inflammation and degradation of systemic glucose metabolism, which ultimately affects systemic metabolism [29]. Triglyceride levels in the liver were lower in mice with WC than in obese controls. The regulation of microRNAs in the hypothalamus may account for the disparities between these studies. WC produced by repeated high-fat diets resulted in weight gain, impaired glucose tolerance, and obesity in most mice [30]. However, it had no long-term effect on mice with high levels of miR-219 in the hypothalamus, suggesting that epigenetic variables like non-coding RNA could be used to treat metabolic disorders and WC [23].

### Intestine barrier and WC

**Fig. 1:**
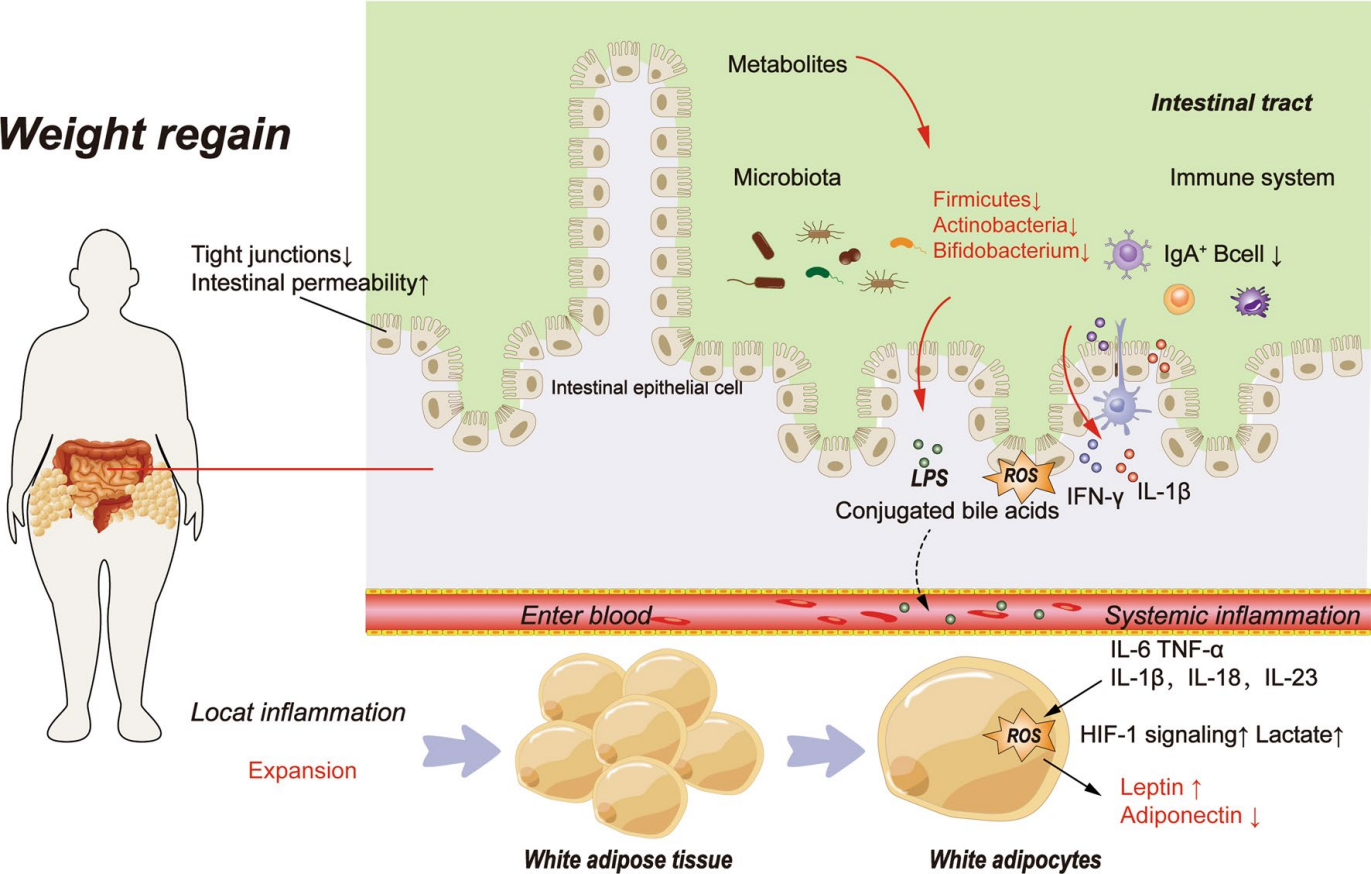
WC results in weight gain, manifested by changes in the intestinal environment resulting in local and systemic inflammation. WR plays a significant role in the formation of WC. It is characterized by alterations in the intestinal environment, specifically the disruption of tight junctions between intestinal epithelial cells and a decrease in intestinal permeability. More metabolic waste enters the intestine at this time, causing changes in the distribution structure of microbiota, as evidenced by a decrease in *firmicutes, actinobacteria,* and *bifidobacteria* and an increase in LPS production. Simultaneously, the intestinal immune system is altered, as evidenced by a decrease in IgA + B cells and an increase in inflammatory factors. IL-1β and IFN-γ levels rise, and more ROS are produced; these substances enter the bloodstream and form systemic metabolism. It also causes expansion of the local white adipose tissue, which leads to a dysregulated endocrine state of the white adipose tissue, increased secretion of inflammatory factors, activation of the inflammatory pathway HIF-1, and elevated leptin and adiponectin decrease in the majority of individuals, resulting in local metaflammation

WR is associated with increased appetite and high intake of a high-fat diet, which causes damage to the gut barrier, including inhibition of mucus production, weakened tight junctions, changes in intestinal villus structures, and intestinal inflammation [31]. It will ultimately impact the endocrine function and immune environment of adipose tissue further (Fig. 1). In addition, changes in the intestinal environment caused by WC, which affect appetite and internal environment homeostasis, cause adipocytes to grow, proliferate, and activate in response to various lipid metabolism disturbances, resulting in the dysregulation of numerous adipocytokines and the accumulation of proinflammatory immune cells [32–34]. These adipokines combine with cytokines and chemokines produced by immune cells to promote inflammation locally [35, 36]. Proinflammatory cytokines, adipokines, and muscle factors may also exacerbate adipose tissue inflammation and promote metaflammation. It creates a vicious cycle that maintains adipose tissue and skeletal muscle inflammation, resulting in complex weight maintenance and simple WR [37, 38]. These alterations subsequently activate inflammatory signals, resulting in the secretion of proinflammatory cytokines in the stomach, the downregulation of intestinal immune defenses, and the exacerbation of inflammation in metabolic organs [39]. Changes in the gut barrier can also affect appetite. It has been discovered that the villus surface area of the jejunum is increased in rats undergoing diet and weight loss. The intestinal absorption area may also be increased, which may be an essential mechanism for promoting appetite, resisting weight loss, inducing WR, and leading to WC [40]. Intestinal inflammation and oxidative stress are anticipated to exacerbate the breakdown of the mucus barrier and increase the cellular permeability of the epithelium. Due to bacterial distribution, pathobiont components and metabolites enter the circulation and extraintestinal tissues via the intestinal lumen (such as adipose tissue). Lipopolysaccharides and other toxins from gut microorganisms enter the bloodstream, resulting in metabolic endotoxemia, which promotes local or systemic inflammation and oxidative stress, leading to metabolic disorders [41–43]. With approximately 55% of operational taxonomic units (OTUs) not returning to normal levels, the dysregulated composition of the gut microbiota during the primary obesity phase does not return to its original composition upon normalization of weight loss. It may be due to the increased growth of flavonoid-metabolizing bacteria, which reduces the quantity of bioavailable flavonoids, thereby promoting WC and negatively regulating energy expenditure mediated by the oxidative stress regulatory protein UCP1 [44].

Although it is still unclear which microbial strains can be targeted to improve WC, many promising avenues have been identified. It has been hypothesized that intestinal microbial-bile acid crosstalk can stimulate WR in mice, resulting in weight circulation. By increasing thermogenesis, treatment with *Parabacteroides distasonis* or non-12α-hydroxylated bile acids improves weight gain [45]. Some researchers have investigated the distribution of intestinal microbiota to prevent weight loss in anti-obesity mouse models and discovered that *Christensenellaceae* is the most significant biomarker for reducing WR, which may represent a potential therapeutic target [46]. In addition, recent research has linked specific microbial strains to weight loss. For instance, the abundance of *Bifidobacterium* in the gastrointestinal tract and *eosinophilic Bifidobacterium* in the mucous membrane is inversely associated with weight loss [47]. In addition, *Firmicutes* and *actinomycetes* produce conjugated linoleic acid, which promotes weight loss by increasing energy expenditure and metabolism, decreasing adipogenesis, promoting lipolysis, and inhibiting adipocyte death [48]. Consequently, some researchers believe that autologous fecal microbiota transplantation (aFMT) can be used to transplant bacteria that promote weight loss and maintain weight during recovery. This treatment effectively maintains lower adipokines and inflammatory markers, such as leptin, C-reactive protein, and interleukin 6 (IL-6). It suggests that aFMT may preserve the metabolic benefits of weight loss and prevent WC; this benefit is associated with lower adipokines and inflammatory markers levels [49].

### Controversial views on the WC-induced metabolic health consequences

There are divergent opinions regarding these potential health risks. The first is the study of the effect of WC on insulin action; prior to 2015, the majority of research focused on the negative effect of WC on the risk of type 2 diabetes [17]. Recent studies based on animal models found that while WC mice exhibited normal systemic insulin action, their glucose tolerance was significantly impaired [30]. Secondly, regarding lipids, some studies have demonstrated that WC does not affect blood lipid levels in mice but only body weight change [50]. In population studies, it was found that HDL-C showed a sustained improvement despite weight recovery [51]. Even if weight is regained after weight loss on a low-calorie diet, there are still beneficial long-term effects, as evidenced by improved liver fat, liver test results, and insulin resistance, according to some research [52]. There is also a divergent opinion regarding inflammatory markers improvements, with lipocalin and IL-6 being maintained for 24 months following weight restoration [53]. Lastly, there is an effect on lifespan, with one study revealing a significant increase in lifespan in WC mice compared to obese mice and similar benefits for mice with sustained moderate weight loss [54]. In population studies with large samples, the effect on lifespan has also been viewed favorably, with deliberate weight loss-induced WC not being associated with an increased risk of death from all causes [55]. However, in these studies: the degree or cycle of WC was inconsistently defined, with most of the above studies cycling once, i.e., weight loss and then WR versus long-term obesity, whereas multiple cycles were more likely to be associated with health risks. In studies of inflammatory marker improvement, comparisons were made with subjects' baseline levels and not those who maintained a healthy weight after weight loss. In population-based research studies of diabetes risk, differences in diabetes risk were not statistically significant. It may account for the discrepancies between the reports' findings.

## Tissue changes in WC: local metaflammation of adipose and muscle tissue

**Fig. 2:**
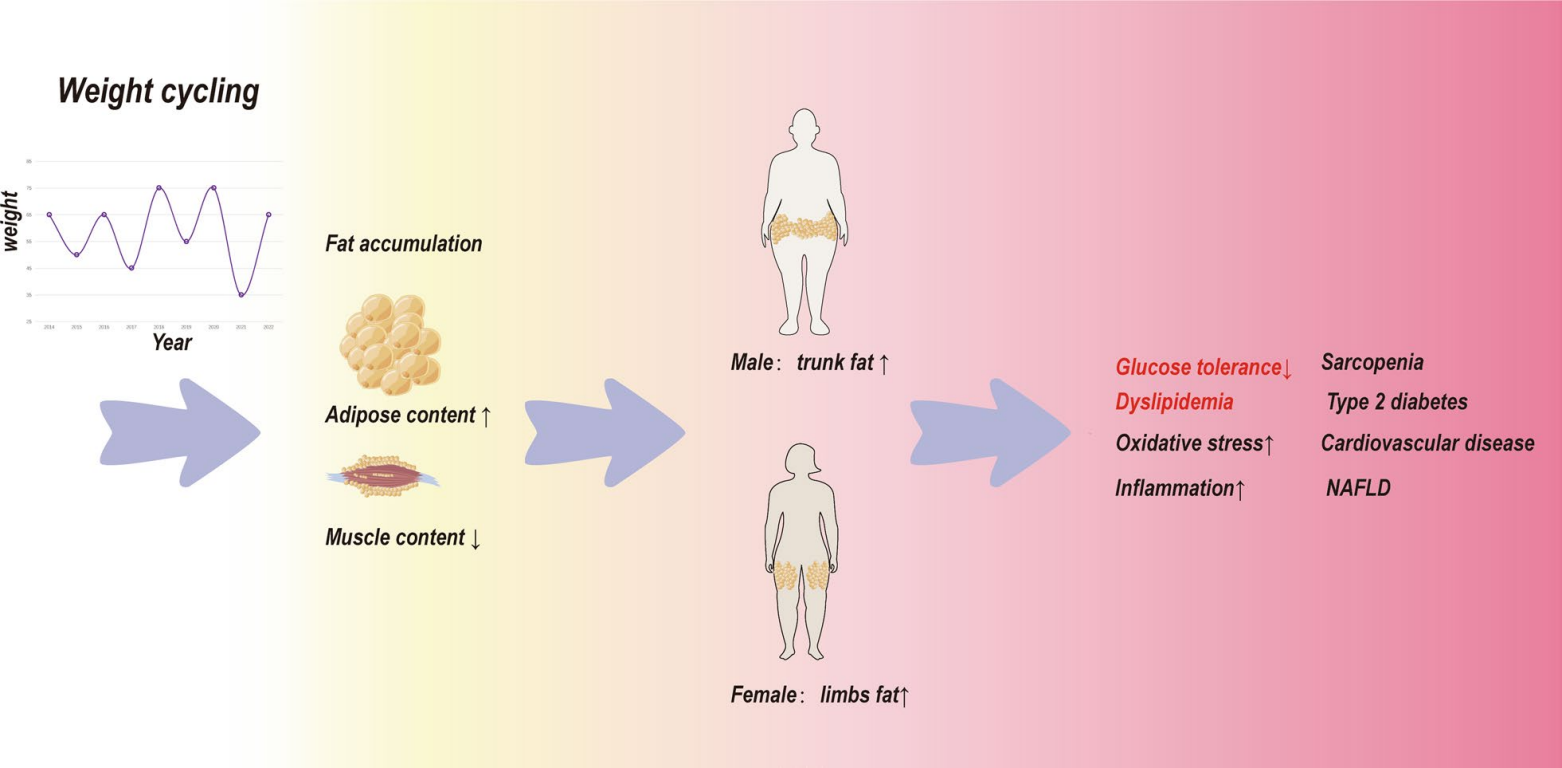
Changes in body composition and metabolism during WC. Many individuals develop WR following weight loss. During WR, adipose tissue increases and muscle tissue decreases, with fat deposition in the trunk predominating in men and fat deposition in the limbs predominating in women. Alternating WL and WR compose the WC. The intrinsic principle of WC is associated with oxidative stress and inflammation-induced metaflammation, which leads to decreased glucose tolerance and dyslipidemia, and ultimately sarcopenia, type 2 diabetes, cardiovascular disease, and NAFLD

During the weight loss phase of WC, significant muscle loss and an inflammatory state are evident [56]. During the WR phase, adipose tissue swells significantly, and the inflammatory state is exacerbated, whereas muscle does not significantly recover, and its local inflammatory state is maintained [16]. Muscle loss and fat accumulation create a vicious cycle that increases metaflammation via a complex interaction between proinflammatory cytokines, oxidative stress, effects on insulin secretion and glucose tolerance, and mitochondrial dysfunction [16, 57] (Fig. 2).

### Adipose issue in WC

Adipose tissue stores more energy than is required to regain initial weight, and as weight is regained, the adipocytes' size and capacity to store lipids gradually increase [22, 58]. The cause of WC may be that overweight or obese individuals frequently use dieting to lose weight but find it challenging to maintain the diet after weight loss and revert to their previous eating habits. However, after a period of caloric restriction, the original diet will be reinstated, leading to WC. Increasing food intake and processing efficiency under the influence of emotion and hormones increase the volume and number of adipocytes, resulting in a decreased metabolic rate and fat accumulation [59]. The cellular pressure of adipose tissue was significantly higher in individuals who regained weight than those who maintained a stable weight. This phenomenon may be associated with the expression of weight-recovery-related genes, such as actin, glucose treatment, and nutritional perception-related genes. Hypoxia-inducible factor-1 (HIF-1), recombinant human galectin-1 (LGALS1), alpha-enolase (ENO1), and activating transcription factor 2 (ATF2) are believed to be essential regulators [60]. When fat cells decrease in size due to weight loss, white adipose tissue adapts by altering its metabolic properties and absorbing great energy. With WC, there is a significant increase in fat storage in the body, which is detrimental to lean body mass. Men are more likely to deposit fat in the trunk, whereas women are more likely to deposit fat in the limbs [61]. According to a study, the cycle of weight gain or loss led to increased food conversion efficiency and changes in body composition in male mice [62]. WC brought on by repeated healthy and unhealthy dietary changes will eventually result in a substantial increase in total body fat, particularly visceral adipose tissue [44, 63].

Moreover, internal fat deposition caused by multiple weight cycles increases dramatically compared to maintaining a healthy diet over the long term [64]. However, this change is a protective physiological phenomenon, and WC performance may adapt the body to store more fat to protect it from potential hunger consumption. Studies have demonstrated that WC decreases resting energy expenditure (REE) and hastens weight recovery, suggesting that adaptations associated with weight loss in REE may impede weight loss and hasten weight recovery [65]. Indicators of this condition include increased dietary requirements for high-calorie and high-fat foods, physical activity levels, and glucose tolerance. In mice, weight loss decreased fat mass and adipocyte size [54]; however, refeeding restored body fat and cell size, contributing to fat deposition [66]. Compared to mice fed a high-fat diet, mice fed the WC paradigm gained weight more rapidly, exhibited more pronounced preadipocyte proliferation, and had more white adipose tissue in the epididymis. Throughout the recurrent phase of WC, adipose tissue inflammation is associated with adipose tissue remodeling, specifically visceral fat remodeling [67]. Adipose tissue can increase to 30–50% of the total body mass in highly obese patients. In extremely obese patients, adipose tissue can account for 30–50% of the total body mass. After regaining weight and returning to obesity, numerous stromal vascular cells and distinct immune cell subgroups exist in obese fat [68, 69]. Active metabolic changes suggest that the expanding, proliferating adipose tissue may also be an immune organ.

### Skeletal muscle in WC

Increased adipose tissue in obese individuals stores lipids that accumulate ectopically in skeletal muscle to form peri-muscular adipose tissue (PMAT) [70]. Excessive adipose tissue deposition within the muscle impairs the structural integrity and function of the muscle, resulting in a dramatic decrease in muscle mass (lean body mass). It is especially true in WC; muscle mass and strength decrease drastically after significant weight loss [71]. It is because muscle mass and the degree of adipose tissue deposition are not fully restored after weight loss, and lean body mass is drastically reduced while total fat content increases, which is especially noticeable in women [72]. In one study, those with WC had a 3.8-fold increased risk of reduced muscle mass, while those with severe WC had a 5.2-fold increased risk of muscular dystrophy [16]. During WR, feedback signals from fat and muscle mass consumption can regulate energy intake and adaptive thermogenesis. In skeletal muscles, WR inhibits the oxidation of dietary fat and downregulates the expression of genes involved in fat metabolism [73]. Fat restoration is also a prominent feature of excessive fat accumulation that precedes the recovery of lean body mass [74]. It may account for changes in body composition following WC [75]. Obesity and sarcopenia are associated with a decreased basal metabolic rate, increased oxidative stress, and metabolic issues [76]. Then it increases the risk of cardiovascular disease, depression, disability, and death [77]. WC causes a change in body composition comparable to sarcopenic obesity(SO) and is believed to be a causative factor in SO [16].

The skeletal muscle is the primary organ in which glucose uptake by glucose transporter protein 4 (GLUT4) is mediated by insulin [78]. The maintenance of skeletal muscle mass plays a critical role in health maintenance by helping to regulate glucose homeostasis. Maintaining skeletal muscle mass is vital for maintaining health by regulating glucose homeostasis. Loss of muscle mass due to intramuscular lipid accumulation is associated with metabolic inflammation, which can cause the development and worsening of impaired glucose tolerance by inhibiting mitochondrial function and increasing the formation of reactive oxygen species, accompanied by increased secretion of proinflammatory cytokines such as tissue necrosis factor alpha (TNF-α) and IL-6, and altering the secretion levels of leptin and resistin [79]. The loss of skeletal muscle mass impairs insulin secretion and glucose tolerance. It increases the release of free fatty acids in adipose tissue, suppressing the growth hormone/insulin growth factor-1 axis and reducing muscle regeneration [80].

## Possible causes of WC: metaflammation in the microenvironment centered on immune cells

WC is frequently associated with the enduring effects of previous obesity, which may be a persistent or even partial amplification of adipose tissue's immune and inflammatory status. Presently, it is believed that adipose tissue is an immune organ with an endocrine function that contains immune cells, such as intrinsic immune cells (e.g., macrophages, neutrophils, dendritic cells, eosinophils, mast cells, and natural killer cells) and adaptive immune cells (e.g., T and B lymphocytes) [81, 82]. The adipokines and inflammatory factors secreted by adipose tissue and immune cells, respectively, promote inflammation and oxidative stress molecular signal activation, thereby initiating metaflammation. Suppose this metaflammation persists for an extended period. In that case, immune cells are transformed into cells with a proinflammatory phenotype and a faster rate of fat deposition, which may be the cellular-molecular mechanism underlying WC.

### Expression of WC inflammation in immune cell phenotype

**Fig. 3:**
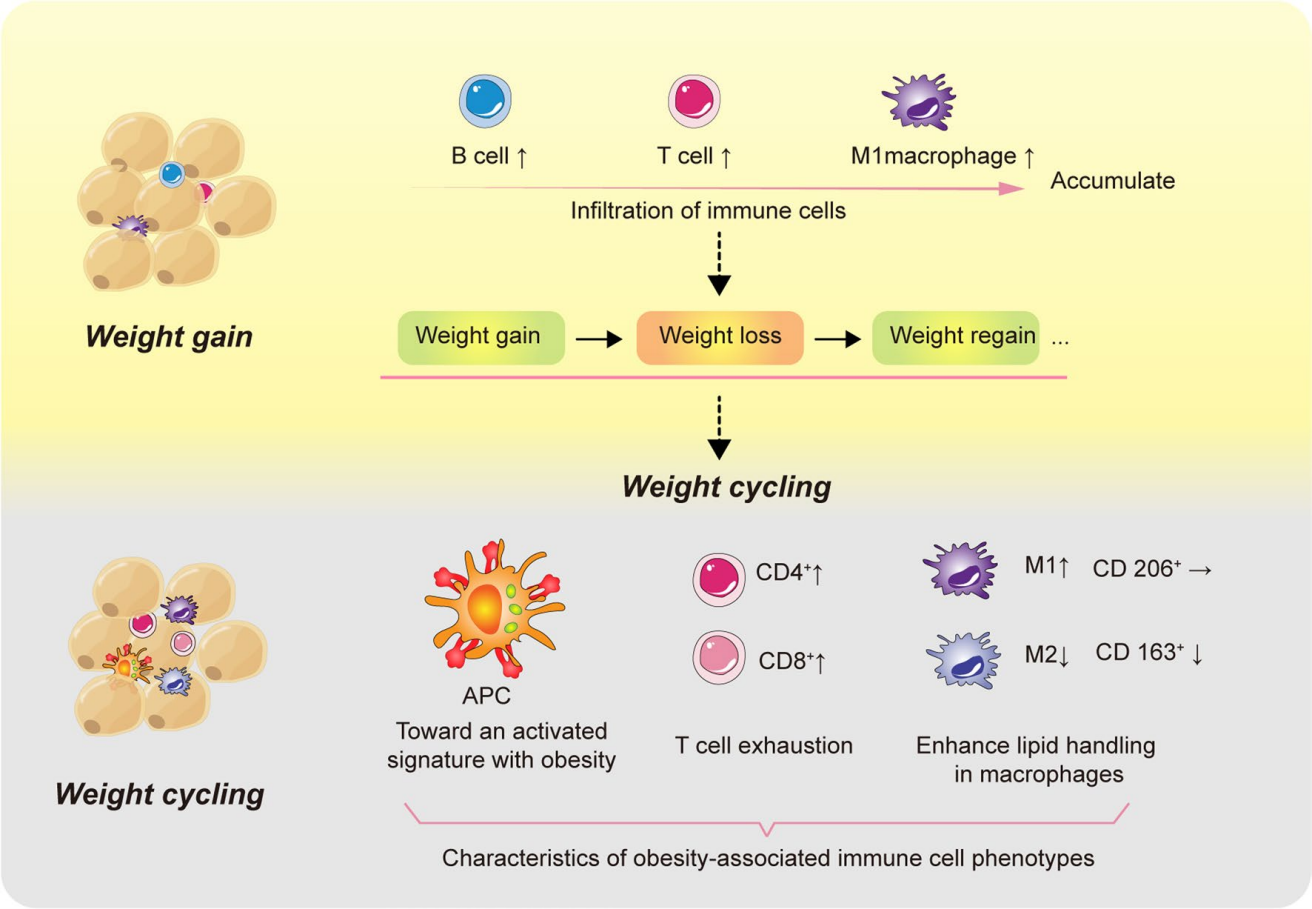
Characteristics of immune cell infiltration and inflammatory cytokine secretion in adipose tissue during weight gain and cycling are depicted. There is a significant infiltration of immune cells, including B cells, T cells, and M1 macrophages, into adipose tissue with weight gain. This repeated weight loss and weight gain will be characterized by an obesity-associated immune cell phenotype with its distinctive characteristics, including APC toward an activated signature with obesity, marked and depleted CD4 and CD8 cell infiltration, and a gradual transformation over time into a macrophage-dominated M1-like inflammatory polarization with decreased M2 polarization

A hyperactive immune response in adipose tissue may result in metabolic abnormalities during WC [83]. Immune cells circulate in the bloodstream and migrate to various tissues, each of which has unique environmental factors. During an immune response, fluctuating nutritional conditions and oxygen availability influence immune cells' temporal and spatial distribution [84]. During WC, the spatial and temporal interactions between immune cells in adipose tissue at the onset and resolution of inflammation are associated with metaflammation (Fig. 3).

The accumulation of immune cells in adipose tissue exacerbates obesity-associated inflammation [85]. B cells are among the first immune cells to accumulate in adipose tissue during weight gain, and they have a significant inflammation-promoting effect on adipose tissue expansion. In contrast, the number of T cells and macrophages does not change significantly. The number of T cells and macrophages gradually increases after that [86]. Weight gain caused by adipose tissue impairment and meta-inflammation can be reduced by systemic B-cell depletion. B cells specific to adipose tissue can release proinflammatory cytokines, such as IL-6 and interferon, and regulate T cell and macrophage activation [87]. During WC, the body maintains a high T-cell and macrophage accumulation. Weight gain may expose antigens in obese adipose tissue, leading to the tissue accumulation of effector and memory T cells. Inflammation of adipose tissue gradually subsides during weight loss; however, memory T cells may remain in the tissue. Consequently, the subsequent weight gain caused by WC permits the re-exposure of obese adipose tissue antigens, which may result in a more effective and rapid secondary immune response [88]. Then the number of CD4^+^ and CD8^+^ T cells increases in adipose tissue, and the expression of various helper T cells and related cytokines increases dramatically, resulting in impaired systemic glucose tolerance. The antigen, which contributes to forming memory T cells in adipose tissue, elicits a robust immune response [89]. In addition, researchers discovered that WC mice regained weight significantly faster, which may be because CD4^+^ T lymphocytes mediate obesity memory variables and result in WC. This type of fat memory can last for at least two months [90]. It may be speculated that excessive adaptive immune responses in adipose tissue may contribute to metabolic dysfunction during WC.

The most abundant immune cells in the adipose tissue of obese individuals are macrophages, the primary immune cells involved in adipose tissue inflammation [91]. They can accumulate in subcutaneous and visceral adipose tissue and are associated with systemic obesity and adipocyte size [92]. M1 and M2 cells are the most prevalent types of macrophages, and various disease states have distinct immune and metabolic characteristics. Obesity is believed to cause M1 macrophage polarization by directly targeting macrophage metabolic processes, such as increases in circulating glucose and fatty acid substrates, lipid toxicity, and tissue hypoxia [93]. Adipose tissue-derived macrophages can produce the proinflammatory cytokines IL-6 and TNF-α, inhibiting lipoprotein lipase, disrupting fat storage in adipose tissue, and phagocytosing necrotic and apoptotic muscle cells [94]. The classical IL-6 signaling pathway activation triggers an anti-inflammatory response in macrophages and a proinflammatory response in T cells [95]. Quantity and polarization of macrophages in adipose tissue are not modulated positively in WC [96]. According to this study, obese mice exhibited a significantly more proinflammatory M1 polarization state than lean mice. However, the number and polarization of adipose tissue macrophages were not regulated by body mass index, and there was no significant change compared to the obesity control group, indicating that body mass index cannot reduce the proinflammatory response of M1 polarization [89]. SThe inflammatory macrophage subset CD206^+^ infiltrates significantly after weight gain (M1 polarization) and persists in adipose tissue during weight change, despite the dietary intervention or medical treatment [97]. The persistence of the inflammatory characteristics of macrophages in WC mice is considered one of the causes of WC.

Recent single-cell sequencing data from weight-cycling mice adipose tissue revealed that obesity-induced immune cell memory in adipose tissue persists even after weight loss and deteriorates gradually as weight is regained. WC results in the maintenance of obesity-related inflammation, including antigen-presenting cell activation, T-cell exhaustion (chronic CD8^+^ T-cell stimulation of antigen presentation may result in T-cell exhaustion), and lipid processing in macrophages. The number and polarization of adipose tissue macrophages did not improve despite the persistence of obesity-induced changes [83]. The fact that macrophages associated with lipids are more inflammatory suggests that the prevention or treatment of WC may begin with the inflammatory maintenance characteristics associated with obesity. These characteristics may represent a type of immune memory that can result in WC-caused metabolic diseases. These characteristics may represent a type of immune memory that can lead to metabolic diseases caused by WC.

### Molecular mechanism

#### Inflammatory biomarker of immune cells in WC

Immune cell pathways are related to immune cell accumulation and cytokine secretion regulation. In response to inflammatory stimuli, receptor-interacting protein kinases (RIPK) 1 and 3 can mediate inflammation, apoptosis, and necrotizing apoptosis as fundamental regulators of inflammatory cell activity. Obesity is associated with single nucleotide polymorphisms (SNPs) in the human RIPK1 gene, which can be inhibited to reduce adipose tissue inflammation and increase invariant natural killer T (iNKT) cell activity during weight gain [98, 99]. Through the participation of TLR2 and TLR4 and the activation of the JNK pathway, a branch of the NF-κB and MAPK pathways, a mixture of palmitic acid or long-chain fatty acids may increase the expression of proinflammatory molecules in macrophages and induce M1 polarization. Palmitic acid and its metabolite ceramide also induce the activation of NLRP3 inflammatory bodies, and the activation of the inflammasome and caspase-1 is further driven by NF-κB and JNK pathways, resulting in the maturation and secretion of proinflammatory cytokines IL-1β and IL-18 and triggering oxidative stress and inflammatory responses [100]. Blocking NLRP3 can ameliorate systemic and local inflammation caused by adipose deposition by reducing the expression of key molecules (e.g., COL1A1, COL4A3, COL6A3, and MMP2) involved in adipose tissue fibrosis induced by LPS [101]. In one study, WC mice's epididymal adipose tissue expressed higher levels of TNFα and IFNγ and similar levels of IL-1β when compared to weight-gain mice without WC. Nonetheless, the expression of macrophage markers and the number of macrophages with crown-like structures increased, indicating an increase in necrotic adipose tissue. Additionally, WC increases total STAT3 and NF-κB pools in liver tissue, which is consistent with the immune status and inflammatory activation in adipose tissue and suggests that inflammatory changes in WC may be systemic [102]. Changes in immune cell populations and adipose tissue gene expression may contribute to the aggravation of metabolic phenotypes during WC. Compared to obese mice, WC mice specifically upregulated specific immune cell receptors and immune response-related genes (*Irgm2 Ikzf3,Cd3g, Cd22,Ccl5, Aif1, Gbp2,Cd83,Rasgrp1,Ly86, C1qc, C1qb, H2-DMB1*). It indicates that activation of adipocyte death-related adaptive immunity in adipose tissue may result in adverse metabolic effects [103]. According to additional research, the immune cell signatures of WC are associated with adipokine signaling. Compared to obese mice, WC mice had significantly increased liver inflammation, macrophage infiltration, and necrosis in adipose tissue. This upregulation is associated with Lmot4-resistin signaling, indicating crosstalk between adipose tissue and liver in WC [104].

#### Metaflammation caused by oxidative stress

Weight increases oxidative stress; therefore, the underlying mechanism of WC may be related to oxidative stresss [105]. Even after weight loss, a history of WC negatively affected 5-hydroxymethyl-2'-deoxyuridine (5-OHmU) levels in mammary DNA in female rats, indicating significant oxidative damage [106]. During rapid growth and expansion of white adipose tissue [107], anoxic regions may form due to inadequate relative oxygen perfusion or increased oxygen consumption. Cellular hypoxia may stimulate the hypoxia-inducible factor-1 (HIF-1)-related pathway, thereby triggering local inflammation [108]. WR can increase adipocyte oxygen consumption and hypoxic conditions, increase HIF-1-related gene expression and lactic acid accumulation, and stimulate the mobilization of adipose tissue from the liver. HIF-1α overexpression causes insulin resistance and adipose tissue inflammation in mouse adipocytes [109]. During the WR phase of WC, the expression of oxidative stress-related genes increased in the subcutaneous adipose tissue of the subjects. HIF-1 is believed to be a key regulator of these genes. *LGALS1*, *ENO1*, and *ATF2* are crucial nodes that increase the risk of WC circulation [60]. When adipose tissue is exposed to hypoxia, many inflammatory factors and inflammation-related adipokines are released. Hypoxia is associated with macrophage infiltration sites and can cause insulin resistance in adipocytes, resulting in adipose tissue fibrosis [110]. A high-fat diet is associated with increased levels of reactive oxygen species (ROS) in colon epithelial cells, activation of the apoptotic pathway, and disruption of the colon epithelial barrier and other colonic environments [111]. Local or systemic inflammation can increase the formation of intracellular ROS, which can result in mitochondrial dysfunction [112]. Metaflammation is closely linked to intestinal barrier dysfunction [113]. ROS has effects not only in the intestine but also in skeletal muscle, where it can inhibit glucose oxidative metabolism and lead to mitochondrial dysfunction [114]. The effect of WC begins with the destruction of the intestinal barrier, followed by the deposition and expansion of adipose tissue in the skeletal muscle, causing local hypoxia and excessive ROS, affecting the function and structure of the skeletal muscle, emphasizing the "dominant position" of fat, and finally resulting in diseases characterized by metastasis.

#### The role of fat factors in metaflammation

Adipose tissue can also function as an endocrine organ to secrete resistin, adiponectin, and adiponectin. Adipokines are cytokines and hormones produced by adipose tissue that influence the polarization of macrophages. In mice, WC affects blood lipids, blood glucose-insulin homeostasis, and adipokine levels and is associated with body mass and adipose tissue remodeling [115]. In the state of WC, the energy demand is excessively high, and the rate of fat deposition is significantly increased, resulting in lipid overload, which is accompanied by lipotoxicity, oxidative stress, and mitochondrial dysfunction [116, 117]. In addition, WC significantly reduced adiponectin's expression and increased resistin's expression, suggesting that it may induce metabolic disorders through the dysregulation of adipokine expression [118]. WC significantly decreased adiponectin expression and increased resistin expression, suggesting that it may induce metabolic disorders through the dysregulation of adipokine expression [53, 104]. It has been observed that dysregulation of the expression of the adipokine C1q/TNF-Related Protein 3 (CTRP3) in adipose tissue and the resulting amplification of inflammation may be the mechanism underlying WC-induced metabolic dysfunction [119].

Leptin is a potent activator of innate and adaptive immune cells during weight gain, causing the release of proinflammatory cytokines and macrophage polarization M1 [120]. In the WC model, fat deposition was accelerated, serum leptin levels decreased by nearly half, and the activity of lipogenic enzymes (fatty acid synthase and malic enzyme) in white adipose tissue increased and remained elevated for many days [121]. In addition, exogenous leptin supplementation has been demonstrated to reduce systemic and intestinal inflammation, which may serve as a starting point for addressing WC [122, 123]. A large amount of resistin is secreted by adipocytes during body WC, which may cause liver inflammation because signal transducer and activator of transcription 3 (stat3) activate the upregulation of proinflammatory genes (*IL-1β, IL-6,* and *IL-8*) [124]. Adiponectin can exert an anti-inflammatory effect and promote the M2 phenotype, but individuals with WC have lower adiponectin levels. Higher circulating adiponectin levels can cause macrophages to resist the stimulation of inflammation, protecting obese mice from glucose intolerance and insulin resistance [125]. Multiple WC results in poor weight loss and easier WR, significantly lower serum and adipose tissue adiponectin levels, and increased expression of resistin, leptin, IL-6, and TNF. Specifically, IL-6 levels remained elevated after three WC [56]. It suggests that WC-related adipokine changes may exacerbate adipose tissue inflammation and chronic systemic inflammation resulting from an imbalance in adipokine production and release.

## Conclusion and future directions

Although significant progress has been made in recent years in the fields of weight loss, treatment of obesity, and obesity-related complications as a result of the development of new drugs and treatment modalities, research on the prevention of WR to prevent the formation of WC is still in its infancy and developing phase. The cause of WC may be due to intractable meta-inflammation in adipose and muscle tissue. This chronic low-grade inflammation causes weight rebound when it spreads throughout the body, resulting in WC and associated metabolic complications. Inhibiting the onset and progression of metabolic inflammation and enhancing the immune microenvironment in adipose and muscle tissue may therefore be the first step in preventing the formation of WC. In this regard, adiposity, as a complex endocrine and immune organ, plays a significant role in many adverse health outcomes associated with WC, as adipokines released from adipose tissue and cytokines released from immune cells interact via inter-organ crosstalk and physiological changes.

There are still many unanswered questions in the field. (1) Although the activation and induction of the immune microenvironment by WC-induced accumulation of adipose tissue have been initially elucidated, the role of immune cells and cytokines in weight restoration remains unclear. (2) The innate or specific immune system's direct targets in obesity-related muscle apoptosis are unknown. (3) Current animal models for WC typically employ male animals, but female rodents exposed to their hormones may exhibit a distinct phenotype and degree of metaflammation. To prevent WC, future research must continue to interfere with metaflammation effects based on adipose-associated immune cells fundamentally. It requires broader and more in-depth studies of immune cell subpopulation distribution in adipose and muscle tissue, even extending to the relationship between stromal cells and immune cells, as well as studies of the distinction between acute inflammatory signaling and metaflammation. Combining larger scale analyses to dissect these relationships better, thereby enabling more precise and targeted techniques such as cell clustering and cell type annotation using the broader CITE-seq panel, enables the discovery of precise interventions for diet-induced WC, metaflammatory activation pathways, antigen receptor diversity, and even the establishment of weight loss prognostic profiles and the use of immune cell spatiotemporal data for further research.

Currently, clinical applications can be investigated from three perspectives: From a systemic perspective, anti-inflammatory diets that reduce metabolic inflammation, in conjunction with energy restriction strategies for conventional weight loss, maybe the optimal nutritional interventions for WC prevention. Exogenous leptin and adiponectin supplementation [123, 126], leucine supplementation (in individuals with low leptin levels) [127], or injections during weight loss to preserve muscle and prevent fat deposition. Specific immune cell agonists, blockers, or targeted blockade of inflammatory metabolic pathways may be considered at the cellular and molecular level to reduce metabolic inflammation [128]. However, this requires consideration of the safety and status of organ factors, and it is essential to conduct human studies because laboratory-based results may differ from clinical trial results. Considering the multifactorial nature of obesity as a disease, parallel to clinical interventions, exercise, lifestyle, and psychosocial health should be investigated.

## Data Availability

Not applicable.
